# Identification of Susceptible Loci and Enriched Pathways for Bipolar II Disorder Using Genome-Wide Association Studies

**DOI:** 10.1093/ijnp/pyw064

**Published:** 2016-07-22

**Authors:** Chung-Feng Kao, Hui-Wen Chen, Hsi-Chung Chen, Jenn-Hwai Yang, Ming-Chyi Huang, Yi-Hang Chiu, Shih-Ku Lin, Ya-Chin Lee, Chih-Min Liu, Li-Chung Chuang, Chien-Hsiun Chen, Jer-Yuarn Wu, Ru-Band Lu, Po-Hsiu Kuo

**Affiliations:** Department of Public Health & Institute of Epidemiology and Preventive Medicine, College of Public Health, National Taiwan University, Taipei, Taiwan (Dr Kao, Mr Lee, and Dr Kuo); Department of Agronomy, College of Agriculture & Natural Resources, National Chung Hsing University, Taichung, Taiwan (Dr Kao); National Center for Genome Medicine, Institute of Biomedical Sciences, Academia Sinica, Taipei, Taiwan (Mrs Chen, Dr Yang, Dr Chen, and Dr Wu); Department of Psychiatry & Center of Sleep Disorders, National Taiwan University Hospital, Taipei, Taiwan (Dr Chen); Department of Nursing, Cardinal Tien Junior College of Healthcare & Management, Yilan, Taiwan (Dr Chuang); Department of Psychiatry, School of Medicine, Taipei Medical University, Taipei, Taiwan (Drs Huang, Chiu, and Lin); Department of Psychiatry, Taipei City Psychiatric Center, Taipei, Taiwan (Dr Huang); Department of Psychiatry, Wan Fang Medical Center, Taipei, Taiwan (Dr Chiu); Department of Psychiatry, Taipei City Hospital and Psychiatric Center, Taipei, Taiwan (Dr Lin); Neurobiology and Cognitive Science Center, National Taiwan University, Taipei, Taiwan (Dr Liu); Department of Psychiatry, National Taiwan University Hospital and College of Medicine, National Taiwan University, Taipei, Taiwan (Dr Liu); Department of Psychiatry, National Cheng Kung University and Hospital, Tainan, Taiwan (Dr Lu); Research Center for Genes, Environment and Human Health, National Taiwan University, Taipei, Taiwan (Dr Kuo).

**Keywords:** genome-wide association, bipolar II disorder, enriched pathways, polygenic score, heritability estimate

## Abstract

**Background:**

This study aimed to identify susceptible loci and enriched pathways for bipolar disorder subtype II.

**Methods:**

We conducted a genome-wide association scan in discovery samples with 189 bipolar disorder subtype II patients and 1773 controls, and replication samples with 283 bipolar disorder subtype II patients and 500 controls in a Taiwanese Han population using Affymetrix Axiom Genome-Wide CHB1 Array. We performed single-marker and gene-based association analyses, as well as calculated polygeneic risk scores for bipolar disorder subtype II. Pathway enrichment analyses were employed to reveal significant biological pathways.

**Results:**

Seven markers were found to be associated with bipolar disorder subtype II in meta-analysis combining both discovery and replication samples (*P*<5.0×10^–6^), including markers in or close to *MYO16*, *HSP90AB3P*, noncoding gene *LOC100507632*, and markers in chromosomes 4 and 10. A novel locus, *ETF1*, was associated with bipolar disorder subtype II (*P*<6.0×10^–3^) in gene-based association tests. Results of risk evaluation demonstrated that higher genetic risk scores were able to distinguish bipolar disorder subtype II patients from healthy controls in both discovery (*P*=3.9×10^–4^~1.0×10^–3^) and replication samples (2.8×10^–4^~1.7×10^–3^). Genetic variance explained by chip markers for bipolar disorder subtype II was substantial in the discovery (55.1%) and replication (60.5%) samples. Moreover, pathways related to neurodevelopmental function, signal transduction, neuronal system, and cell adhesion molecules were significantly associated with bipolar disorder subtype II.

**Conclusion:**

We reported novel susceptible loci for pure bipolar subtype II disorder that is less addressed in the literature. Future studies are needed to confirm the roles of these loci for bipolar disorder subtype II.

## Introduction

Bipolar disorder (BP) is a severe mental illness characterized by mood swings between mania and depression. The lifetime prevalence of BP was reported with a range from 0.8% to 4% in the general population (Kessler et al., 2005; Kato, 2007), with great disease burden worldwide (Whiteford et al., 2013). There are 2 main distinct subtypes of BP, bipolar I (BP-I) and bipolar II (BP-II) disorders, according to the Diagnostic and Statistical Manual of Mental Disorders-IV (DSM-IV) diagnostic system (American Psychiatric Association, 1994). The 2 subtypes demonstrated quite different clinical features, such as episode frequency, comorbidity, family history, etc. (Kukopulos et al., 1980; Coryell et al., 1984; Savino et al., 1993; Pini et al., 1997). For example, BP-II patients tend to experience more evening tiredness and shorter inter-episode well intervals than BP-I patients (Judd et al., 2003; Chung et al., 2012). Moreover, patients with BP-I or BP-II can be distinguished by abnormally functional differences in ventral striatal activity during reward anticipation (Caseras et al., 2013), suggesting distinct neural mechanisms predisposing to mood dysregulation between the BP-I and BP-II groups. Other differences are also reported, such as cerebral glucose metabolism (Ketter et al., 2001), and neurobiological functioning (e.g., the fronto-limbic metabolism and executive function) in brain regions (Li et al., 2012). These observations altogether imply that there might be distinct etiology and genetic mechanisms underlying different subtypes of BP.

With a high heritability estimated around 80% for BP (McGuffin et al., 2003), a strong genetic component is consistently reported by family, twin, and adoption studies, as well as molecular genetic studies (please refer to a review article by Barnett and Smoller, 2009). In particular, large scale genome-wide association (GWA) studies are commonly employed in the last decade to search susceptibility loci for complex human traits (McCarthy et al., 2008). Several GWA studies have identified many loci for the risk of developing BP (The Wellcome Trust Case Control Consortium, 2007; Baum et al., 2008; Ferreira et al., 2008; Sklar et al., 2008; Lee et al., 2011; Kuo et al., 2014; Muhleisen et al., 2014; Xu et al., 2014); however, conclusive and replicated findings are relatively scattered. Some examples are the *ANK3* and *CACNA1C* genes (Ferreira et al., 2008; Muhleisen et al., 2014). To identify disease-associated genetic variants, one of the crucial steps is to define phenotypically homogeneous patient groups. However, patients in previous GWA studies of BP are usually a mixture of the BP-I and BP-II subgroups, with a few focused on only the BP-I subtype (Baum et al., 2008; Sklar et al., 2008; Lee et al., 2011; Kuo et al., 2014) but none solely on the BP-II subtype. The BP-II subtype is relatively understudied in many regards.

One important feature of BP is its high comorbidity with other mental disorders. According to the National Comorbidity Survey Replication study, 90% of BP patients cooccurred with other psychiatric disorders, especially commonly seen anxiety and substance use disorders (Merikangas et al., 2007; Tsai et al., 2012), such as alcohol dependence (Mitchell et al., 2007). Comorbid conditions bring in phenotypic heterogeneity and further complicated the search of genetic risk factors for BP. Due to the nature of phenotypic and etiological complexity in BP, the underlying pathogenesis is not yet fully understood. There is a strong need to minimize phenotypic noises in study samples to better decipher the genetic basis of the less studied BP-II subtype. In our Taiwanese Han BP-II samples, relatively low comorbidities were observed for anxiety and substance use disorders compared with those in Caucasian samples (McElroy et al., 2001; Merikangas et al., 2007), suggesting less concern about the confounding effects of comorbidities in searching for genetic factors.

The present study aimed to identify susceptibility loci for BP-II by conducting a GWA study in the Taiwanese Han population. A replication study using an independent sample was employed to confirm the associated genetic variants identified for BP-II in the discovery stage. Using polygenic genetic risk prediction score (GRS), we demonstrated that the selected markers are capable of separating BP-II patients from healthy controls. Moreover, we estimated the variance explained by single nucleotide polymorphisms (SNPs) on the whole genome level for BP-II in the current study, the so-called chip heritability. We also conducted functional pathway analysis to describe the biological profiles underlying etiological mechanisms of BP-II using the GWA dataset.

## Methods

### Discovery and Replication Samples

A total of 189 unrelated BP-II patients, including 84 males (44.4%) and 105 females (55.6%), was consecutively recruited from several psychiatric clinics in Taiwan during 2008 and 2012. These index probands, aged between 18 and 70 years old, were clinically diagnosed by psychiatrists according to the DSM-IV (American Psychiatric Association, 1994). Patients with schizophrenia, schizoaffective, or substance-induced mood disorders were excluded from this study. Demographic and clinical features were obtained by well-trained interviewers using the Composite International Diagnostic Interview (Kessler and Ustün, 2004) and the Chinese version of the modified Schedule of Affective Disorder and Schizophrenia-Lifetime (Endicott and Spitzer, 1978). The concordance for BP diagnosis between the Composite International Diagnostic Interview and DSM-IV was excellent, with a kappa coefficient of 0.94 (Merikangas et al., 2007). More detailed information of patients’ ascertainment and interview procedures were described elsewhere (Lai et al., 2010; Tsai et al., 2012). In our BP-II patients, 24 subjects and 16 subjects had comorbidity with anxiety and substance use disorders, respectively, indicating a lower degree of phenotypic heterogeneity than patients in most of the previous GWA studies in Caucasian samples (Merikangas et al., 2007; Sublette et al., 2009). This study was approved by the institutional review board of all the participating hospitals. All participants provided written informed consent.

Healthy controls were randomly selected from a pool of 3380 healthy controls recruited from the community subjects of the Han Chinese Cell and Genome Bank (HCCGB) in Taiwan. The recruitment details were documented elsewhere (Pan et al., 2011). In brief, controls were recruited from 329 nonaboriginal township or city districts in Taiwan using a stratified, 3-stage clustering sampling design. Of the 3380 controls, 1773 controls (nearly equal proportions of males and females) with a Taiwanese Han ancestry, who were found to have no definite diagnosis of any major medical or mental illnesses, underwent genotyping at the genome-wide level and were treated as controls in this sample. Written informed consent was obtained from all participants. In total, the discovery samples consisted of 189 unrelated BP-II patients and 1773 healthy controls.

To conduct a replication study for the initial findings, we recruited 283 additional unrelated BP-II patients from psychiatric clinics in Taiwan. All participants were evaluated with an interview by an attending psychiatrist and followed by a more detailed interview with a clinical psychologist using the Chinese version of modified Schedule of Affective Disorder and Schizophrenia-Lifetime to determine DSM-IV diagnoses, which has good inter-rater reliability as aforementioned. For more detailed information regarding ascertainment and measurement, please refer to Huang et al. (2004). Patients with major mental illnesses other than BP (e.g., borderline personality disorder, drug dependence, and cognitive disorders) were excluded. We used the 2-day minimum for hypomania in the diagnosis of BP-II. In the replication samples, there were no subjects with comorbidity of either anxiety or substance use disorders. Similarly, healthy controls were from the HCCGB. We randomly selected 500 healthy controls without overlapping with the 1773 controls in the discovery samples. All participants provided written informed consent. The replication samples were used to confirm initial findings, which included association analysis (i.e., single-marker and gene-based association tests), risk evaluation, and heritability estimate.

### Genotyping Data and Quality Controls

Genomic DNA was extracted from peripheral blood using the Puregene DNA isolation kit (Gentra Systems, Minneapolis, MN). We used the Affymetrix (San Francisco, CA) Axiom Genome-Wide CHB1 Array Plate for both the discovery and replication samples, and genotyping was conducted at the National Center for Genome Medicine, Academia Sinica, Taiwan. Genotype calling was performed using the standard procedure with the default parameters suggested by the platform manufacturer. Genotyping validation was performed using the Sequenom iPLEX assay (Sequenom MassARRAY system; Sequenom, San Diego, CA) at National Center for Genome Medicine. For the discovery samples, a total of 1962 subjects was genotyped with 628132 SNPs. Similarly, 783 subjects were genotyped in the replication samples. The genotyping call rate was 98.8% for all subjects.

Quality control procedures were done firstly with each individual, including sample quality, kinship, and population stratification. We used Dish sample quality control (DQC) to monitor nonpolymorphic locations to specify signal and background channels. DQC is the recommended quality control metric for Axiom genome-wide arrays in genotyping console software (http://www.mscience.com.au/upload/pages/axiom/axiom_bos1_faq.pdf) where the genomic sequence/allele is known. We discarded 3 subjects whose DQC values were not satisfactory. Additionally, 15 subjects were excluded due to the overall call rate <97%. We also computed plate pass rate (samples passing DQC and 97% call rate divided by total samples on the plate) to check plate-wise genotyping biases. Samples with plate pass rate >95% and average call rate of passing sample >99% were retained in the analysis. No sample was removed during this step. Second, we checked inbreeding coefficient and identity by state, so that samples with strong kinship were eliminated. In total, 56 individuals (8 BP-II patients and 48 healthy controls) were removed from the discovery samples due to having similarity measures far away from the clustering (supplementary Figure 1). Third, we used multidimensional scaling analysis on the genome-wide identity by state pairwise distance to eliminate 8 samples, which were outliers away from the clustering on the scatter plot (supplementary Figure 2). As a result, 1880 subjects, including 181 BP-II patients and 1699 healthy controls were retained (details please refer to supplementary Table 1). For the replication samples, following the same quality control procedures for individuals, 12 BP-II subjects were removed from the replication samples, resulting in a total of 771 subjects, including 271 BP-II patients and 500 healthy controls in the following analyses.

We also performed quality control procedures for markers. We removed markers if they failed Hardy-Weinberg tests with *P* < .0001, genotype missing rate >5%, minor allele frequency (MAF) < 0.05, or had bad calling in clustering. We also compared B allele frequency of 2 groups (i.e., cases vs the whole sample, controls vs the whole sample) to remove markers whose difference in B allele frequency was >2%. As a result, a total of 557169 SNPs in the discovery samples, and 559196 SNPs in the replication samples were retained for imputation.

### Imputation and Gene Mapping

Imputation was carried out using IMPUTE2 v3 (Howie et al., 2009), with haplotype reference panels released in March/April 2012 from the 1000 Genomes Project on the basis of HapMap build 37 (https://mathgen.stats.ox.ac.uk/impute/data_download_1000G_phase1_integrated_SHAPEIT2.html). Only imputed SNPs with high genotype information content (i.e., IMPUTE info score >0.5) were used in association analyses. In total, 29495 018 SNPs and 29488 193 SNPs were imputed with high confidence for each individual in the discovery and replication samples, respectively. Following the same quality control procedures for markers, 4222038 SNPs and 4209 610 SNPs were retained in the discovery and replication samples for analyses, respectively. Using 20kb upstream and downstream of the gene boundaries, each of the 4222 038 SNPs (discovery samples) and 4209 610 SNPs (replication samples) were all mapped into 17619 protein-coding genes. The genomic inflation factor was 0.98 in the discovery samples (supplementary Figure 3) and 1.01 in the replication samples under additive genetic model, indicating less concern about population structure and genotyping error.

### Genetic Association Analyses

We performed single-marker association and gene-based association tests for BP-II. Logistic regression analysis was performed with an additive genetic model. All models were adjusted for gender and age to correct for different distributions in gender and age across BP-II patients and healthy controls. SNPs with *P* < 5×10^–6^, combined from the discovery and the replication samples, were considered to be suggestive and reported in single-marker association analysis.

We conducted gene-based association analysis using PLINK. To reduce computational load arises from gene-level analysis in the scale of whole genome with 50000 permutations, we conducted a multi-stage analysis to obtain a gene-level empirical significance estimation (Liu et al., 2010). Information from a set of SNPs (association *P* < .1) within a gene was aggregated. To account for linkage disequilibrium (LD) among markers, only SNPs having *r* ^2^<0.5 with each other were retained for each gene. We also fitted logistic regression models to perform multi-stage gene-based association tests. Empirical *P* values were calculated with at most 50000 permutations. We first conducted standard gene-based analysis using PLINK with 100 permutations for the whole genome. If a gene with resulting *P* ≤ .1, then 500 permutations were performed for the selected genes. If a gene with resulting *P* ≤ .02, then 2000 permutations were performed for the selected genes. If a gene with resulting *P* ≤ .005, then 50000 permutations were performed for the selected genes. We reported genes with *P* ≤ 5×10^–4^ in the discovery samples.

Finally, to combine results from the discovery and replication samples, meta-analysis was applied. We used the inverse Gamma model with a shape parameter (α) of 0.1 (Zaykin et al., 2007) to summarize association information across the 2 samples.

### Risk Evaluation

Recently, we proposed a GRS method (Kao et al., 2014) and successfully applied it to evaluate the risk information in a prioritized candidate-gene set of BP. Markers in weak LD (*r* ^2^<0.2) with each other were selected to calculate GRS. We further utilized 3 thresholds (association *P* < .001, .005, and .01) to prune the SNPs for risk evaluation. Markers were selected from the discovery samples, and the distributions of GRS between BP-II patients and healthy controls were then compared in both the discovery and replication samples in the present study. We used the Wilcoxon rank-sum test to examine the distributions of GRS between BP-II patients and healthy controls.

### Heritability Estimate

We conducted the genome-wide complex trait analysis (GCTA) via the restricted maximum likelihood to estimate the variance explained by all the SNPs on the whole genome scale for BP-II (Yang et al., 2011). In brief, we first calculated the genetic relationship matrix (GRM) between any of 2 individuals from genome-wide SNPs. For each pair of individuals, we discarded one individual whose relationship in GRM is greater than a cutoff value of 0.025. As a result, a total of 1880 and 771 unrelated individuals from the discovery and the replication samples were retained to estimate genetic variance explained by SNPs in the chip. We fitted a mixed model using the GRM on a binary disease trait to estimate the variance explained by genome-wide SNPs. All models were corrected for the prediction error derived from the MAF of causal variants to obtain an unbiased estimate of heritability. We specified a range of disease prevalence (i.e., 0.01, 0.02, 0.03, 0.04, and 0.05) to transform the estimated heritability from the observed scale to liability scale. The settings of prevalence were based on prior prevalence estimation for BP in the general population (Kessler et al., 2005; Kato, 2007).

### Pathway-Based Enrichment Analysis

We conducted pathway analyses to examine the common processes and biological mechanisms for BP-II using the identified susceptible genes. The Kyoto Encyclopedia of Genes and Genomes (KEGG), BioCarta, Reactome, and Gene Ontology (GO) terms were used for functional annotation and enrichment analyses. In total, 2784 pathways were analyzed. A total of 452 genes having *P* < .01 in gene-based association analysis or the genes with at least one SNP having *P* < .001 in single-marker association analysis were selected for pathway analysis. Hypergeometric test was performed using the web-based Gene Set Enrichment Analysis (http://www.broadinstitute.org/gsea/index.jsp) and a pathway with a *P* < 5×10^–3^ after Bonferroni correction was considered significant.

## Results

In single-marker association analyses, 25 SNPs with odds ratios ranging from 1.70 to 2.57 were reported to have suggestive signals, which are listed in Table 1. Of these, 8 markers were mapped to 4 known genes, including *KCNAB2* (supplementary Figure 4), *MYO16* (supplementary Figure 5), *NTRK3* (supplementary Figure 6), and *LOC100507632* (supplementary Figure 7). The former 3 are coding genes. *LOC100507632*, located in chromosome 8, is a gene coding for a long intergenic RNA. In addition, there were 4 SNPs located nearby 2 gene regions, *HSP90AB3P* (rs77034375, rs3828554, and rs62318301) and *FLRT2* (rs61212586). The remaining 13 SNPs were not mapped to any gene regions. Among the 25 SNPs listed in Table 1, 7 of them were replicated (highlighted in gray in Table 1) with a combined *P* ≤ 5.0×10^–6^, including 2 SNPs in *MYO16* (rs9583266, *P* = 2.3×10^–6^; rs2182501, *P* = 3.3×10^–6^), 3 SNPs close to *HSP90AB3P*(rs77034375, *P* = 2.5×10^–7^; rs3828554, *P* = 2.2×10^–7^; rs62318301, *P* = 5.4×10^–7^), 1 SNP in chromosome 4 (rs114640425, *P* = 9.6×10^–7^), and another SNP in chromosome 10 (rs10887909, *P* = 7.8×10^–7^). Detailed information of the 7 replicated markers is shown in supplementary Table 2. Results of gene-based association analyses are listed in Table 2. Ten genes showed suggestive signals (*P* < 5.0×10^–4^), while only one gene, *ETF1*, was replicated with a gene-based *P* value of 0.006.

**Table 1.** Results of Single Marker Associations for Bipolar II Disorder

SNP	CHR: Position	Mapped Gene	Minor/Major Allele	Chip / Impute	Discovery Samples			Replication Samples			Meta-Analysis *P* Value
					MAF (case/control)	*P* Value	OR	MAF (case/control)	*P* Value	OR	
rs3789559	1:6105109	*KCNAB2*	T/C	Chip	0.425/0.314	2.1×10^–6^	1.76	0.320/0.318	0.92	1.01	5.5×10^–6^
rs3789560	1:6104715	*KCNAB2*	G/A	Impute	0.427/0.317	2.4×10^–6^	1.77	0.322/0.322	0.98	1.00	6.2×10^–6^
rs3789556	1:6108863	*KCNAB2*	G/A	Impute	0.415/0.300	2.5×10^–6^	1.78	0.306/0.308	0.97	0.99	6.5×10^–6^
rs77034375	4:88815986	Nearby *HSP90AB3P*	T/C	Impute	0.124/0.062	1.9×10^–6^	2.53	0.096/0.057	7.0×10^–3^	1.76	**6.8×10** ^**–7**^
rs3828554	4:88813875	Nearby *HSP90AB3P*	C/T	Impute	0.121/0.060	2.9×10^–6^	2.53	0.096/0.055	4.0×10^–3^	1.84	**6.6×10** ^**–7**^
rs62318301	4:88811182	Nearby *HSP90AB3P*	G/A	Impute	0.119/0.059	4.9×10^–6^	2.49	0.093/0.055	6.0×10^–3^	1.79	**1.5×10** ^**–6**^
rs114640425	4:37761675		G/C	Impute	0.143/0.075	3.2×10^–6^	2.49	0.103/0.066	1.7×10^–2^	1.60	**2.2×10** ^**–6**^
rs6991165	8:57435745	*LOC100507632* (*LINC00968*)	A/G	Chip	0.481/0.353	4.8×10^–6^	1.70	0.373/0.374	0.96	0.99	1.2×10^–5^
rs10887909	10:90857775		A/G	Impute	0.438/0.326	2.9×10^–6^	1.73	0.384/0.320	1.5×10^–2^	1.32	**1.8×10** ^**–6**^
rs9583266	13:109250314	*MYO16*	A/G	Impute	0.195/0.105	1.7×10^–6^	2.06	0.140/0.109	8.0×10^–2^	1.35	**3.0×10** ^**–6**^
rs2182501	13:109255438	*MYO16*	C/T	Impute	0.196/0.108	3.3×10^–6^	2.01	0.143/0.109	6.0×10^–2^	1.38	**5.0×10** ^**–6**^
rs66581286	13:109259587	*MYO16*	A/G	Impute	0.193/0.106	3.6×10^–6^	2.01	0.137/0.106	0.09	1.34	6.7×10^–6^
rs11619281	13:109225931		G/A	Impute	0.231/0.138	3.6×10^–6^	2.27	0.179/0.150	0.14	1.25	7.9×10^–6^
rs34497953	13:109226359		C/T	Impute	0.231/0.138	3.6×10^–6^	2.27	0.179/0.150	0.14	1.25	7.9×10^–6^
rs35218525	13:109179086		G/A	Impute	0.243/0.148	4.0×10^–6^	1.89	0.187/0.161	0.19	1.22	9.5×10^–6^
rs11616644	13:109169847		C/T	Impute	0.232/0.139	4.0×10^–6^	1.90	0.187/0.155	0.11	1.27	8.0×10^–6^
rs11616603	13:109171415		G/A	Impute	0.236/0.142	4.0×10^–6^	1.89	0.188/0.158	0.13	1.25	8.5×10^–6^
rs11616630	13:109171673		C/A	Impute	0.236/0.143	4.1×10^–6^	1.89	0.188/0.158	0.13	1.25	8.7×10^–6^
rs4771589	13:109182384		T/G	Impute	0.243/0.149	4.2×10^–6^	1.89	0.187/0.161	0.19	1.22	9.9×10^–6^
rs71439369	13:109196377		T/C	Impute	0.237/0.143	4.6×10^–6^	1.88	0.183/0.160	0.25	1.19	1.1×10^–5^
rs1924345	13:109189032		C/T	Impute	0.243/0.149	4.6×10^–6^	1.88	0.191/0.162	0.14	1.24	1.0×10^–5^
rs1924348	13:109191801		G/A	Impute	0.236/0.143	4.8×10^–6^	1.88	0.188/0.159	0.14	1.25	1.0×10^–5^
rs2147148	13:109190538		G/A	Impute	0.236/0.143	4.8×10^–6^	1.88	0.188/0.159	0.14	1.25	1.0×10^–5^
rs61212586	14:86123937	Nearby *FLRT2*	A/G	Impute	0.275/0.163	4.6×10^–6^	1.94	0.150/0.153	0.84	0.97	1.2×10^–5^
rs16941261	15: 88655520	*NTRK3*	C/G	Impute	0.129/0.069	3.9×10^–6^	2.57	0.068/0.070	0.87	0.96	1.0×10^–5^

Abbreviations: CHR, chromosome; MAF, minor allele frequency; SNP, single nucleotide polymorphism.

Association analyses using logistic regression with an additive model while adjusted for gender and age.

*P* values in meta-analysis were calculated based on the inverse gamma method and we reported SNPs (highlighted in gray) with combined *P* value <5×10^–6^.

**Table 2.** Results of Gene-Based Association Analyses for Bipolar II Disorder

Gene	Number of SNPs within a Gene		Number of SNPs Having *P* < .1		Number of SNPs Having *P* < .1 and *r*2<0.5		Empirical *P* Value	
	Discovery Samples	Replication Samples	Discovery Samples	Replication Samples	Discovery Samples	Replication Samples	Discovery Samples	Replication Samples
*AL355490.1*	59	58	1	1	1	1	2.0×10^–5^	.25
*RRP12*	190	182	4	3	1	1	2.0×10^–5^	.28
*KCNAB2*	40	40	7	0	2	0	1.0×10^–4^	1
*TLL2*	377	375	2	6	1	2	1.2×10^–4^	.89
*PJA2*	42	37	1	2	1	1	3.6×10^–4^	.33
*VSIG10*	126	111	12	6	1	2	3.8×10^–4^	.41
*CDKL4*	78	75	66	8	1	1	4.2×10^–4^	.15
*IWS1*	204	198	111	17	3	2	4.4×10^–4^	.36
*MAP4K3*	203	194	160	6	1	1	4.8×10^–4^	.46
*ETF1*	52	51	38	50	1	1	4.9×10^–4^	6.0×10^–3^

We reported genes with empirical *P* < 5×10^–4^ based on 50,000 permutations in the discovery samples. All models were adjusted for gender and age. Gene highlighted in gray was successfully replicated in the replication samples.

Comparisons of GRS distributions between BP-II and healthy controls are displayed in Table 3. For the discovery/replication samples, a total of 1144/1097, 6046/5876 and 13023/11416 independent markers were retained for GRS analyses, which corresponded to different association thresholds at *P*<.001, .005, and .01, respectively. Our results showed that BP-II patients had significantly higher GRS than controls at the 3 thresholds, and *P* values ranged from 3.9×10^–4^ to 1.0×10^–3^ for the discovery samples and 2.8×10^–4^ to 1.7×10^–3^ for the replication samples. Therefore, the selected marker sets from the discovery samples had the capability to distinguish BP-II patients from healthy controls not only in the discovery samples but also in the replication samples using the GRS method.

**Table 3.** Results of the Genetic Risk Score (GRS) in the Discovery and Replication Samples

			Difference Test					
			Discovery Samples			Replication Samples		
Threshold at *P* Value (P*)	No. of SNPs Having *P*< P*		No. of SNPs Having *P*< P*& Low LD (*r*2<0.2)		*P* Value	No. of SNPs Having *P*< P* & Low LD (*r*2<0.2)		*P* Value
.001	1302		1144		1.0×10^–3^	1097		1.7×10^–3^
.005	6706		6046		3.9×10^–4^	5876		2.8×10^–4^
.01	13023		11735		1.5×10^–3^	11416		2.0×10^–3^

*P* Value was calculated using Wilcoxon rank-sum test to examine different distributions of GRS between BP-II patients and healthy controls.

Table 4 showed heritability estimated from all SNPs for BP-II in the discovery and replication samples. Only SNPs with MAF≥0.05 were retained for heritability estimates. The phenotypic variances explained by these SNPs for BP-II ranged from 35.85% to 55.11% in the discovery samples (*P*=9.2×10^–5^), and 39.33% to 60.47% in the replication samples (*P*=1.8×10^–2^), given the prevalence of BP-II equals 1~5%.

**Table 4.** Estimation of Variance Explained by Genome-Wide SNPs

Sample	Genetic Variance, Vg (s.e.)	Residual Variance, Ve (s.e.)	Phenotypic Variance, Vp (s.e.)	Heritability Estimate, *h*2 (s.e.)					
				Not Adjusted by Disease Prevalence	Adjusted by Disease Prevalence				
					0.01	0.02	0.03	0.04	0.05
Discovery	0.0684 (0.019)	0.0938 (0.019)	0.1622 (0.005)	0.4217 (0.119)	0.3585 (0.101)	0.4258 (0.120)	0.4753 (0.134)	0.5160 (0.146)	0.5511 (0.156)
Replication	0.1481 (0.074)	0.0798 (0.073)	0.2279 (0.012)	0.6498 (0.320)	0.3933 (0.193)	0.4671 (0.230)	0.5214 (0.256)	0.5660 (0.279)	0.6047 (0.297)

*h* ^2^ =Vg/ Vp represents the estimate of variance explained by the genome-wide SNPs on the observed scale. Adjusted *h* ^2^ represents the estimate of variance explained by genome-wide SNPs, taking into account the prevalence of bipolar II disorder from the general population.

Results of the pathway analyses for BP-II are displayed in Table 5. Twenty-four biological pathways were associated with BP-II. The top 7 pathways are neurological system process, transmembrane transporter activity, apoptosis, programmed cell death, developmental biology, neuronal system, and gastrin CREB signaling pathway via PKC and MAPK. These pathways are involved with neurodevelopmental function, signal transduction, and immune and inflammation response, suggesting their roles in the pathological mechanisms underlying BP-II.

**Table 5.** Results of the Pathway Enrichment Analysis for Bipolar II Disorder

Pathway	No. Genes in Gene-Set	# Genes Overlapping with Our Gene List	*P* Value	Adjusted *P* Value
Neurological system process (GO)	379	18	3.4×10^–8^	1.2×10^–5^
Transmembrane transporter activity (GO)	375	17	1.6×10^–7^	3.1×10^–5^
Apoptosis (GO)	431	17	1.1×10^–6^	1.6×10^–4^
Programmed cell death (GO)	432	17	1.1×10^–6^	1.6×10^–4^
Developmental biology (Reactome)	396	16	1.6×10^–6^	1.9×10^–4^
Neuronal system (Reactome)	279	13	3.3×10^–6^	3.3×10^–4^
Gastrin CREB signaling pathway via PKC and MAPK (Reactome)	205	11	5.0×10^–6^	4.6×10^–4^
Regulation of apoptosis (GO)	341	14	6.1×10^–6^	5.1×10^–4^
Axon guidance (Reactome)	251	12	6.1×10^–6^	5.1×10^–4^
Regulation of development process (GO)	440	16	6.2×10^–6^	5.1×10^–4^
Regulation of programmed cell death (GO)	342	14	6.3×10^–6^	5.1×10^–4^
Substrate specific transporter activity (GO)	392	15	6.6×10^–6^	5.2×10^–4^
Substrate specific transmembrane transporter activity (GO)	344	14	6.7×10^–6^	5.2×10^–4^
G alpha Q signaling events (Reactome)	184	10	1.2×10^–5^	8.8×10^–4^
Transmission across chemical synapses (Reactome)	186	10	1.3×10^–5^	9.4×10^–4^
Sensory perception (GO)	190	10	1.6×10^–5^	1.1×10^–3^
Ion transmembrane transporter activity (GO)	278	12	1.7×10^–5^	1.2×10^–3^
Ligase activity (GO)	97	7	4.1×10^–5^	2.4×10^–3^
Cell adhesion molecules (CAMs) (KEGG)	134	8	4.6×10^–5^	2.6×10^–3^
MAPK signaling pathway (KEGG)	267	11	5.8×10^–5^	3.2×10^–3^
Neuroactive ligand receptor interaction (KEGG)	272	11	6.8×10^–5^	3.7×10^–3^
Arrhythmogenic right ventricular cardiomyopathy (ARVC) (KEGG)	76	6	8.9×10^–5^	4.5×10^–3^
Nervous system development (GO)	385	13	9.6×10^–5^	4.7×10^–3^
Ion channel activity (GO)	149	8	9.7×10^–5^	4.7×10^–3^

Abbreviations: GO, Gene Ontology terms; KEGG, Kyoto Encyclopedia of Genes and Genomes.

*P* values were calculated using hypergeometric test; adjusted *p*-values were computed using the Bonferroni correction. We reported pathways with *P* < 5×10^–3^ after Bonferroni correction. Our gene list consists of 452 genes that selected from gene-based association analysis (only genes with *P* < .01) and single-marker association analysis (only genes with at least one SNP having *P* < .001) for pathway analysis.

## Discussion

This study reported results of the first GWA study focusing on BP-II in the Taiwanese Han population. In addition to a pure patient group with BP-II diagnosis, one specific feature in our samples is low comorbidity rate with other psychiatric disorders. Patients with BP are reported to have high comorbidity with other psychiatric disorders, especially anxiety and subsutance use disorders (Grabski et al., 2008). Such high comorbidities may confound the results and further complicate our interpretation to the genetic findings. The BP-II patients in our discovery samples had a low comorbidity rate in either anxiety or subsutance use disorders, and patients with either comorbid condition were excluded from the replication samples. Therefore, our patients are phenotypically more homogeneous, and the genetic findings are less likely to be confounded by comorbidity status. On the other hand, the sample size in this study is relatively moderate compared with previous large-scale consortium-based GWA studies in psychiatric disorders. We estimated power using oberved effect sizes and MAFs from reported loci (Table 1) assuming disease prevalence of 1%~5%, and the power varies widely in different marker settings, ranging from 0.24 to 0.81. Consequently, results reported in the present study are all within the suggestive range but did not reach genome-wide statistical significance level (Duggal et al., 2008). Despite limited power, some replication attempts were successful using an independent sample of BP-II.

We reported 7 replicated susceptible loci of BP-II (i.e., rs77034375, rs3828554, rs62318301, rs114640425, rs10887909, rs9583266, and rs2182501). These markers are mapped to *MYO16* gene or close to *HSP90AB3P* gene. In addition, a few loci are located in regions of noncoding transcripts. *MYO16* is expressed predominantly in hippocampal neurons to regulate synaptic cytoskeleton in neurocognitive developmental disorders (Liu et al., 2015). Previous evidence suggested that common variants within the *MYO16* gene contribute to the genetic liability of schizophrenia (Rodriguez-Murillo et al., 2014). It is known that schizophrenia and BP might share some common pathophysiology (Konopaske et al., 2014). Thus, genetic findings within this gene may not be specific to BP-II but a more common genetic liability to severe psychiatric disorders. On the other hand, we could not find any hint between *HSP90AB3P* and BP in the literature. Further studies are needed to explore its role in BP-II.

Moreover, to explore the potential roles of the seven markers as expression quantitative trait locus (eQTL), we used HaploReg (http://compbio.mit.edu/HaploReg) (Ward and Kellis, 2012) to search gene regulation databases, and results are shown in supplementary Table 4. We found that only rs10887909 exhibited direct eQTL effects (in total 3 hits) in regulating expressions of *FAS* and *STAMBPL1*|*AL157394*.15|*ACTA2* in blood and subcutaneous tissues. The other 6 markers do not act as eQTLs, but markers in high LD (*r* ^2^≥0.8) with them are predicted to be eQTLs, which regulated several gene expressions, including *PKD2* (blood tissue), *RELL1* (lung tissue), *PGM2* (dendritic cells), *FAS* (blood tissue), *STAMBPL1*|*AL157394*.15|*ACTA2* (subcutaneous tissue), and *HOXA5* (peripheral blood monocytes tissue). Some of these genes were previously reported as susceptible loci for brain or neuro-related traits. For instance, *FAS* gene was reported to be associated with neurotic degeneration in brain volume of Alzheimer’s patients (Erten-Lyons et al., 2009). *STAMBPL1 g*ene regulates Tax trafficking and NF-κB activation in neuroinflammation process (Lavorgna and Harhaj, 2012). *PKD2* gene was reported as a risk locus for handedness and brain asymmetry that have been linked to neurodevelopmental disorders such as dyslexia (Brandler and Paracchini, 2013).

Many psychiatric disorders demonstrate strong connection with immune dysregulation (Wang et al., 2015). Previous studies showed that epithelial cells in foreskin, breast and lung play roles in upregulating potent immunogenic cytokines or chemokines to induce an immune response (Gunther et al., 2009; Slight et al., 2013). Interestingly, we found that rs6991165 (*P*=1.2×10^–5^) in *LOC100507632* gene was associated with BP-I. This SNP is mapped to a long intergenic noncoding RNA (lincRNA: LINC00968) with 7 known transcripts. The biological functions of *LOC100507632* gene in human are not yet clear. We checked the noncoding RNA database of GR.h37.75 (hg19) version from ENSEMBL (http://www.ensembl.org/index.html) and found that this lincRNA tends to express more in epithelial cells, including foreskin (3.981 fragments per kilobase of transcript per million mapped reads [FPKM]), breast (1.323 FPKM), and lung (0.865 FPKM) tissues. The *LOC100507632* gene may play roles in BP-II through immune-related system.

In gene-based association analyses, we reported 10 susceptible loci to be associated with BP-II in the discovery samples. Among them, only one gene, *ETF1* (eukaryotic translation termination factor 1) was replicated (*P*=.006). Replication of this finding in an independent sample increased its credibility. We also found that all overlapping markers in *ETF1* gene in the discovery and replication samples are in the same association directions. The *ETF1* gene, located in the 5q31, plays important roles in regulating the activity in translational termination process and transcriptional regulation (Guenet et al., 1999). We considered that the associations of *ETF1* gene are more specific to BP-II but not with other psychiatric disorders, which benefit from more homogeneous phenotypic presentations in our samples. Using the publically available Psychiatric Genomics Consortium (PGC) GWA data of BP, major depressive disorder (MDD), and schizophrenia (SCZ), we found no association for markers in the *ETF1* gene with all of these traits. In particular, the PGC-BP GWA data consist of mainly BP-I (84%) and fewer BP-II (11%) patients in their discovery samples (Psychiatric GWAS Consortium Bipolar Disorder Working Group, 2011). Their genetic findings are thus mainly attributed to BP-I but not BP-II subtype. Hence, we tend to consider that the *ETF1* gene is a novel and specific susceptible locus for the risk of developing BP-II in the Taiwanese population. Other than the *ETF1* gene, several other genes maybe also interesting targets for BP-II, in particular the *KCNAB2* gene. Although the association is not replicated in an independent sample, markers in this gene also exhibited associations with BP-II in single-marker analyses. The *KCNAB2* gene encodes for β-subunit of the voltage-gated K^+^ channel. Lack of the β-subunit reduces K^+^ channel-mediated membrane repolarization and increases neuronal excitability, which are related to the development of seizures (Heilstedt et al., 2001). Seizure-related disorders and their subsequent behavioral symptoms caused by dyfunctions of the central nervous system are often linked with psychiatric disorders (Tucker, 1998; Tellez-Zenteno et al., 2007), providing clues for the roles of *KCNAB2* in neurological and psychiatric disorders.

Compared with genetic findings in previous GWA studies of BP or BP-I (The Wellcome Trust Case Control Consortium, 2007; Baum et al., 2008; Ferreira et al., 2008; Lee et al., 2011; Kuo et al., 2014; Muhleisen et al., 2014), there are no overlapping genes reported in this GWA study of BP-II. Previous susceptibility loci included *ANK3*, *ODZ4*, *DGKH*, *SP8*, *ST8SIA2*, *KCTD12*, *CACNB2*, *KCNH7*, *MYST4*, *NRXN3*, *SEMA3D*, *PALB2*, *ITIH3*, *CACNA1C*, among others. However, none of these genes showed *P* < 5.0×10^–4^ in the current study. This may suggest that many of the previously reported loci are BP-I specific or the common causes for BP in general. On the other hand, among the 10 associated genes reported in Table 2 in the discovery samples, 3 genes are ever reported to be associated with BP or BP-I in candidate gene association studies, including *RRP12* (Byrne et al., 2014), *TLL2* (de Mooij-van Malsen et al., 2013), and *PJA2* (Ryan et al., 2006) genes.

Polygenic risk scores are commonly adopted in prior GWA studies for the discovery samples (Middeldorp et al., 2011; Whalley et al., 2012). One previous study calculated GRS in subtypes of BP using a sample of 669 subjects (255 BP patients and 414 controls). Aminoff et al. (2015) reported significantly higher scores for the BP patients than controls but no differences between the two BP subtypes (BP-I versus BP-II) based on the polygenic architecture. In the current study, our GRS analysis demonstrated clear seperation between BP-II patients and healthy controls in the discovery samples, and these results further validated in the replication samples. Therefore, the GRS derived from our discovery samples are able to predict BP-II in the replication samples. Due to a certain degree of the genetic overlapping across psychiatric disorders (Bulik-Sullivan et al., 2015a), it is of interest to know whether loci identified from previous GWA studies of other psychiatric disorders could be used to predict BP-II. Similarly, we used PGC GWA data of BP, MDD, and SCZ for the analyses, and results are displayed in supplementary Table 5. In brief, associated loci identified from these GWA datasets could not predict the risk of BP-II in either the discovery or replication samples, except for PGC-SCZ, which showed some predictability for the risk of BP-II in the discovery samples (*P*=.008~.03). However, we need to explain these results with caution. The PGC GWA datasets consisted of mainly Caucasian populations, which are different from our Taiwanese samples. Additionally, PGC-BP data consisted of mainly BP-I subtype rather than BP-II patients, which may limit the predictability of PGC-BP data on BP-II subtype. Alternatively, we could calculate cross-trait LD score (Bulik-Sullivan et al., 2015a, 2015b) to estimate genetic correlations between BP-II and PGC BP/MDD/SCZ datasets. We found that the genetic correlation is the highest for PGC-SCZ (0.50), followed by PGC-BP (0.39), and PGC-MDD (0.37) (results are in supplementary Figure 8), which is consistent with the GRS analyses results. In the future, in addition to genetic data, to include more clinical variables, such as family history or symptom characteristics may further enhance our ability to make good prediciton for complex disorders like BP-II.

We calculated the variance explained by SNPs using the GCTA method. With common genome-wide SNPs, the estimated heritability was up to 55.1% in the discovery samples and 60.5 % in the replication samples in the present study. One previous study reported that the heritability of BP-II is 0.58 in a Norwegian sample (Edvardsen et al., 2008), which is quite close to the heritability estimation using GCTA in the current study. However, if we used a selected marker set, such as SNPs with *P* values less than a preset threshold, the variance explained was down to 17~39% (3~12%) in the discovery samples (replication samples) using 1144~3023 (1097~11416) independent markers (supplementary Table 3), which echoes prior observations that most of the heritability may not be detected due to a lack of power and small effect size of individual SNPs.

We reported 24 enriched pathways for BP-II, including 4 pathways from KEGG, 6 pathways from Reactome, and 14 pathways from GO terms. In addition to commonly studied pathways such as developmental biology and neurological system process, the cell adhesion molecule pathway is the most significant one in the KEGG database. The process of binding with other cells or extracellular matrix is important in neuronal activity and immune system and inflammation response, and may contribute to psychiatric phenotypes (Rosen, 2004; Ohtsubo and Marth, 2006). For instance, it is reported that neuronal cell adhesions are relevant to synaptic formation and neurotransmission at glutamatergic and GABAergic synapses in schizophrenia and BP (O’Dushlaine et al., 2011), which suggest the roles of cell adhesion molecules in BP (Muhleisen et al., 2014). The identified pathway of the neurological system process is also an important one. Previously, we reported 233 prioritized candidate-genes for BP (namely BPDgenes) from multi-dimensional data sources (Kao et al., 2014). There are 17 overlapping genes (*CLRN1*, *DRD4*, *GCH1*, *GRIA1*, *GRIK1*, *GRIN2B*, *GRM1*, *HTR3B*, *HTR6*, *KCNMA1*, *KCNQ3*, *KLK8*, *NOVA1*, *SLC6A4*, *SYN3*, and *TGFB2*) between the BPDgenes and genes in this pathway. Modulation of dopamine (*DRD4*), serotonergic (*SLC6A4*, *HTR3B*), glutamate signaling and regulation (*GRIN2B*), and ion channel (*KCNQ3*) are all important candidates in previous genetic studies of BP and subtypes of BP (Hammer et al., 2012). For instance, *SLC6A4* is reported to be associated with BP-II in a Taiwanese sample (Wang et al., 2014). In addition, a recent gene expression study reported that epigenetic variation in *KCNQ3* may result in dysregulated ion channel function, leading to neuronal hyperexcitability and thus potentially contributing to the pathophysiology of BP (Kaminsky et al., 2015).

There are some limitations in the current study. First, our limited sample size due to the difficulty in diagnosis and recruitment of BP-II patients might result in low power to detect loci with less effect. In general, BP-II is considered to be more difficult to diagnose reliably than BP-I (Andreasen et al., 1981). We estimated that the power to detect loci in our meta-analysis with the observed effect sizes is modest, ranging from 0.24 to 0.81 given the disease prevalence of 1%~5%. Further replication studies in a larger sample are needed to confirm our findings. Second, our pathway analyses relied on the accuracy and completeness of pathway annotation databases. We used 5 commonly adopted annotations, including KEGG, BioCarta, Reactome, canonical pathways, and GO terms. Among our selected 452 genes, only 437 genes were entered for pathway analyses. Some genes that may have potential impacts on BP-II but are not annotated in pathway databases were excluded from our analyses, and thus may sligtly bias the pathway results. For example, the *KAT6B* gene (also known as *MYST4*, located at 10q22.2) was reported as a candidate gene for BP-I (Kuo et al., 2014) but not in any of the pathway databases. Other datasets, such as curated gene sets and positional gene sets, can be considered in future analysis. Lastly, we estimated the variance explained by selected SNPs on the whole genome level for BP-II; however, some BP-II related loci might not be included, such as rare variants. Large-scale GWA studies of BP-II or deep sequencing studies can assist to enhance our understanding about the pathogenesis of BP-II.

In summary, we reported a few risk loci for BP-II in the current study. To our best knowledge, this is the first GWA study soley focused on BP-II. We suggest that *ETF1* is a novel candidate gene for BP-II, and markers that mapped to *MYO16* and *KCNAB2* or close to *HSP90AB3P*, and non-coding gene *LOC100507632* are also potential targets for BP-II. Many previously reported loci of bipolar disorder in the literature might be BP-I specific. We also showed that neurodevelopmental function, signal transduction, neuronal system, and cell adhesion molecules pathways are invovled in the development of BP-II. Further studies and experiments are needed to confirm our findings.

## Statement of Interest

None.

## Supplementary Material

supplementary Figure 1
