# Supplementary material for: Identification of Susceptible Loci and Enriched Pathways for Bipolar II Disorder Using Genome-Wide Association Studies
Source: Int J Neuropsychopharmacol. 2016 Jul 22;19(12):pyw064. doi: 10.1093/ijnp/pyw064 (PMC5203756; doi:10.1093/ijnp/pyw064)
Supplement: supplementary Figure 1 [file Supporting_information_IntJNP_16_0021.doc]

**Supporting information**

**Table S1. Quality control details for the discover and replication samples**

|  | Original samples | DQC<0.82 | Call rate <97% | Plate QC check | Kinship | Population stratification |
| --- | --- | --- | --- | --- | --- | --- |
| **Discovery sample** | | | | | | |
| Healthy controls | 1773 | 1770 | 1755 | 1755 | 1707 | 1699 |
| BP-II cases | 189 | 189 | 189 | 189 | 181 | 181 |
| Total | 1962 | 1959 | 1944 | 1944 | 1888 | 1880 |
| **Replication sample** | | | | | | |
| Healthy controls | 500 | 500 | 500 | 500 | 500 | 500 |
| BP-II cases | 283 | 283 | 283 | 283 | 271 | 271 |
| Total | 783 | 783 | 783 | 783 | 771 | 771 |
| Abbreviation: DQC, Dish sample quality control; QC, quality control. BP-II, bipolar II disorder. | | | | | | |

**Table S2. Summary information of associated markers in the discover samples**

| SNP | CHR: Position | Minor/Major  allele | Sample size | Genotypes (%) | | | Call rate  (%) | MAF | HWE *p*-value |
| --- | --- | --- | --- | --- | --- | --- | --- | --- | --- |
|  |  |  |  | Homo. rare | Het. | Homo. common |  |  |  |
| **Discovery sample:** | | | | | | | | | |
| rs77034375 | 4:88815986 | T/C | 1862 | 0.24 | 12.81 | 86.96 | 99.16 | 0.066 | 0.16 |
| rs3828554 | 4:88813875 | C/T | 1862 | 0.19 | 12.57 | 87.24 | 99.16 | 0.065 | 0.10 |
| rs62318301 | 4:88811182 | G/A | 1846 | 0.19 | 12.29 | 87.52 | 98.41 | 0.063 | 0.14 |
| rs114640425 | 4:37761675 | G/C | 1841 | 0.62 | 15.23 | 84.15 | 98.17 | 0.082 | 0.88 |
| rs10887909 | 10:90857775 | A/G | 1851 | 11.73 | 43.85 | 44.42 | 98.64 | 0.337 | 0.41 |
| rs9583266 | 13:109250314 | A/G | 1843 | 1.76 | 19.69 | 78.54 | 98.27 | 0.116 | 0.07 |
| rs2182501 | 13:109255438 | C/T | 1859 | 1.75 | 20.11 | 78.14 | 99.02 | 0.118 | 0.12 |
| **Replication sample:** | | | | | | | | | |
| rs77034375 | 4:88815986 | T/C | 769 | 0.26 | 13.78 | 85.96 | 99.61 | 0.072 | 0.42 |
| rs3828554 | 4:88813875 | C/T | 767 | 0.26 | 13.56 | 86.18 | 99.35 | 0.070 | 0.58 |
| rs62318301 | 4:88811182 | G/A | 761 | 0.26 | 13.27 | 88.47 | 98.58 | 0.069 | 0.57 |
| rs114640425 | 4:37761675 | G/C | 760 | 0.66 | 14.60 | 84.74 | 98.45 | 0.080 | 0.81 |
| rs10887909 | 10:90857775 | A/G | 763 | 12.06 | 44.56 | 43.38 | 98.83 | 0.343 | 0.75 |
| rs9583266 | 13:109250314 | A/G | 754 | 0.93 | 21.88 | 77.19 | 97.67 | 0.119 | 0.29 |
| rs2182501 | 13:109255438 | C/T | 758 | 1.06 | 21.77 | 77.18 | 98.19 | 0.119 | 0.39 |
| Abbreviation: SNP, single nucleotide polymorphism; CHR, chromosome; MAF, minor allele frequency; HWE, Hardy-Weinberg equilibrium; Homo. rare, homogeneity rare; Het., heterogeneity; Homo. common, homogeneity common.  Note: Analyses were based on 1880 subjects (181 BP-II, 1699 healthy controls) in the discovery samples and 771 (271 BP-II, 500 healthy controls) in the replication samples. | | | | | | | | | |

**Table S3. Estimation of variance explained by genome-wide** SNPs

|  | Training sample | | | | |  | Replication sample | | | | | |
| --- | --- | --- | --- | --- | --- | --- | --- | --- | --- | --- | --- | --- |
| Prevalence | Genetic variance, Vg (s.e.) | Residual variance, Ve (s.e.) | Phenotypic variance, Vp (s.e.) | Heritability,  *h*^2^ (s.e.) | Adjusted *h*^2^ |  | Genetic variance, Vg (s.e.) | Residual variance, Ve (s.e.) | Phenotypic variance, Vp (s.e.) | Heritability,  *h*^2^ (s.e.)^a^ | Adjusted *h*^2 b^ | |
| ***Association p-value<0.01*** | | | | | | | | | | | | |
| 0.01 | 0.0466 | 0.1098 | 0.1564 | 0.2977 | 0.2531 |  | 0.0292 | 0.2001 | 0.2293 | 0.1275 | | 0.0772 |
| 0.05 | 0.0466 | 0.1098 | 0.1564 | 0.2977 | 0.3892 |  | 0.0292 | 0.2001 | 0.2293 | 0.1275 | | 0.1186 |
| ***Association p-value<0.005*** | | | | | | | | | | | | |
| 0.01 | 0.0457 | 0.1189 | 0.1646 | 0.2776 | 0.2360 |  | 0.0197 | 0.2093 | 0.2290 | 0.0858 | | 0.0520 |
| 0.05 | 0.0457 | 0.1189 | 0.1646 | 0.2776 | 0.3628 |  | 0.0197 | 0.2093 | 0.2290 | 0.0858 | | 0.0799 |
| ***Association p-value<0.001*** | | | | | | | | | | | | |
| 0.01 | 0.0340 | 0.1394 | 0.1734 | 0.1959 | 0.1665 |  | 0.0107 | 0.2184 | 0.2291 | 0.0468 | | 0.0284 |
| 0.05 | 0.0340 | 0.1394 | 0.1734 | 0.1959 | 0.2560 |  | 0.0107 | 0.2184 | 0.2291 | 0.0468 | | 0.0436 |
| ^a^ Vg/ Vp represents the estimate of variance explained by the SNPs on the observed scale. ^b^ Adjusted *h*^2^ represents the estimate of variance explained by the SNPs, taking into account the prevalence of bipolar disorder from a general population. | | | | | | | | | | | | |

**Table S4. Summary of eQTL prediction**

**(a) Query SNPs: rs77034375, rs3828554, rs62318301 and variants with r^2^≥0.8 and eQTL hits**

| chr | pos | LD (r^2^) | LD (D’) | variant | Ref | Alt | EUR freq | GERP cons | SiPhy cons | Promoter histone marks | Enhancer histone marks | DNAse | Proteins bound | Motifs changed | GRASP QTL hits | Selected eQTL hits | GENCODE genes | RefSeq genes | dbSNP func annot |
| --- | --- | --- | --- | --- | --- | --- | --- | --- | --- | --- | --- | --- | --- | --- | --- | --- | --- | --- | --- |
| 4 | 87873532 | 0.86 | 0.97 | rs2199557 | A | G | 0.27 |  |  |  |  |  |  | CEBPB, Mrg1::Hoxa9, NF-I, Pou5f1, TLX1::NFIC |  | 1 hit | 27kb 3' of MEPE | 18kb 5' of HSP90AB3P |  |
| 4 | 87875948 | 0.86 | 0.97 | [rs12510860](http://www.broadinstitute.org/mammals/haploreg/detail_v4.1.php?query=&id=rs12510860) | A | G | 0.27 |  |  |  |  |  |  | CACD, Foxp1, Irf |  | 1 hit | 29kb 3' of MEPE | 16kb 5' of HSP90AB3P |  |
| 4 | 87882729 | 0.88 | 0.99 | [rs17714695](http://www.broadinstitute.org/mammals/haploreg/detail_v4.1.php?query=&id=rs17714695) | G | C | 0.27 |  |  |  | FAT, BLD, STRM, VAS, PANC, SPLN, BRN, BONE |  |  | SZF1-1 |  | 1 hit | 36kb 3' of MEPE | 9.1kb 5' of HSP90AB3P |  |
| 4 | 87882887 | 0.88 | 0.99 | rs10222708 | T | C | 0.27 |  |  |  |  |  |  |  |  | 1 hit | 36kb 3' of MEPE | 9kb 5' of HSP90AB3P |  |
| 4 | 87883129 | 0.88 | 0.99 | rs10222997 | G | A | 0.27 |  |  |  |  | LNG |  | CDP, Hand1, Pax-2 | 1 hit | 1 hit | 36kb 3' of MEPE | 8.7kb 5' of HSP90AB3P |  |
| 4 | 87883163 | 0.99 | 0.99 | rs10222816 | A | G | 0.27 |  |  |  |  | LNG |  | Cdx, HNF1, Nkx2, Pou2f2 |  | 1 hit | 36kb 3' of MEPE | 8.7kb 5' of HSP90AB3P |  |
| 4 | 87890030 | 0.98 | 1 | **rs62318301** | A | G | 0.28 |  |  |  |  |  |  | LUN-1, SP2, Smad |  |  | 43kb 3' of MEPE | 1.8kb 5' of HSP90AB3P |  |
| 4 | 87892723 | 1 | 1 | [**rs3828554**](http://www.broadinstitute.org/mammals/haploreg/detail_v4.1.php?query=&id=rs3828554) | T | C | 0.28 |  |  |  |  |  |  | AP-1, ATF3, Cphx, E2F, HEY1, Jundm2, Myc, Pax-3, Pax-4, TATA |  |  | 46kb 3' of MEPE | HSP90AB3P | intronic |
| 4 | 87894834 | 1 | 1 | [**rs77034375**](http://www.broadinstitute.org/mammals/haploreg/detail_v4.1.php?query=&id=rs77034375) | C | T | 0.28 |  |  |  |  |  |  | Pou1f1, TEF-1 |  |  | 48kb 3' of MEPE | 818kb 3' of HSP90AB3P |  |

**(b) Query SNP: rs114640425 and variants with r^2^≥**0.8 and eQTL hits

| chr | pos | LD (r^2^) | LD (D’) | variant | Ref | Alt | EUR freq | GERP cons | SiPhy cons | Promoter histone marks | Enhancer histone marks | DNAse | Proteins bound | Motifs changed | GRASP QTL hits | Selected eQTL hits | GENCODE genes | RefSeq genes | dbSNP func annot |
| --- | --- | --- | --- | --- | --- | --- | --- | --- | --- | --- | --- | --- | --- | --- | --- | --- | --- | --- | --- |
| 4 | 37758427 | 1 | 1 | rs17496030 | T | C | 0.12 |  |  | BLD | ESDR, FAT, STRM, BRST, BLD, SKIN, LIV, GI, ADRL, HRT, KID, LNG, THYM, BONE | ESDR, BRST, BLD, SKIN, ADRL, HRT, LNG, MUS, PLCNT, GI, THYM, OVRY, PANC, MUS, LIV, BRST, MUS, VAS, BLD, BRN, SKIN | CEBPB, FOXA1, P300, POL2, STAT3, CMYC, MAX | Foxp1, Pou3f2 | 1 hit |  | 58kb 5' of Y_RNA | 68kb 5' of PGM2 |  |
| 4 | 37760053 | 1 | 1 | **rs114640425** | C | G | 0.12 |  |  |  | BLD, GI, PANC |  |  | Pax-5 |  |  | 60kb 5' of Y_RNA | 67kb 5' of PGM2 |  |
| 4 | 37761264 | 0.98 | 0.99 | rs17578219 | G | A | 0.12 |  |  | BLD | FAT, BLD, GI, HRT |  |  | GR, Sox |  | 1 hit | 61kb 5' of Y_RNA | 65kb 5' of PGM2 |  |

**(c) Query SNP: rs**[**10887909**](http://www.broadinstitute.org/mammals/haploreg/detail_v4.1.php?query=&id=rs10887909) **and variants with r^2^≥0.8 and eQTL hits**

| chr | pos | LD (r^2^) | LD (D’) | variant | Ref | Alt | EUR freq | GERP cons | SiPhy cons | Promoter histone marks | Enhancer histone marks | DNAse | Proteins bound | Motifs changed | GRASP QTL hits | Selected eQTL hits | GENCODE genes | RefSeq genes | dbSNP func annot |
| --- | --- | --- | --- | --- | --- | --- | --- | --- | --- | --- | --- | --- | --- | --- | --- | --- | --- | --- | --- |
| 10 | 89088319 | 0.81 | 0.97 | [rs10618076](http://www.broadinstitute.org/mammals/haploreg/detail_v4.1.php?query=&id=rs10618076) | ATAG | A | 0.22 |  |  |  |  |  |  | Mef2, Nanog |  | 1 hit | 25kb 5' of MIR4679-1 | 25kb 5' of MIR4679-2 |  |
| 10 | 89089947 | 0.81 | 0.97 | [rs7897422](http://www.broadinstitute.org/mammals/haploreg/detail_v4.1.php?query=&id=rs7897422) | T | C | 0.22 |  |  |  | FAT, SKIN, LNG |  |  | BCL, Glis2, Ik-1, ZBTB7A, Zfp740, Zfx |  | 1 hit | 27kb 5' of MIR4679-1 | 27kb 5' of MIR4679-2 |  |
| 10 | 89097087 | 0.86 | 1 | [rs10887908](http://www.broadinstitute.org/mammals/haploreg/detail_v4.1.php?query=&id=rs10887908) | G | A | 0.22 |  |  |  |  | IPSC |  | CTCF, Nanog |  | 4 hits | 34kb 5' of MIR4679-1 | 34kb 5' of MIR4679-2 |  |
| 10 | 89098018 | 1 | 1 | [**rs10887909**](http://www.broadinstitute.org/mammals/haploreg/detail_v4.1.php?query=&id=rs10887909) | A | G | 0.19 |  |  |  |  |  |  | CEBPB, DMRT2, Mef2, p300 |  | 3 hits | 35kb 5' of MIR4679-1 | 35kb 5' of MIR4679-2 |  |

**(d) Query SNPs: rs9583266,** **rs2182501 and variants with r^2^≥0.8 and eQTL hit**

| chr | pos | LD (r^2^) | LD (D’) | variant | Ref | Alt | EUR freq | GERP cons | SiPhy cons | Promoter histone marks | Enhancer histone marks | DNAse | Proteins bound | Motifs changed | GRASP QTL hits | Selected eQTL hits | GENCODE genes | RefSeq genes | dbSNP func annot |
| --- | --- | --- | --- | --- | --- | --- | --- | --- | --- | --- | --- | --- | --- | --- | --- | --- | --- | --- | --- |
| 13 | 108597966 | 1 | 1 | [**rs9583266**](http://www.broadinstitute.org/mammals/haploreg/detail_v4.1.php?query=&id=rs9583266) | G | A | 0.19 |  |  |  |  | ESDR |  | Maf, Nkx2 |  |  | MYO16 | MYO16 | intronic |
| 13 | 108603090 | 0.99 | 1 | [**rs2182501**](http://www.broadinstitute.org/mammals/haploreg/detail_v4.1.php?query=&id=rs2182501) | T | C | 0.19 |  |  |  |  |  |  | Sox, TATA |  |  | MYO16 | MYO16 | intronic |
| 13 | 108605878 | 0.99 | 1 | [rs9587628](http://www.broadinstitute.org/mammals/haploreg/detail_v4.1.php?query=&id=rs9587628) | G | C | 0.19 |  |  |  |  |  |  |  |  | 1 hit | MYO16 | MYO16 | intronic |

**Table S5. Results of predicting the risk of BP-II using PGC data with different disorders**

|  | Discovery samples | | | | | | | |  | Replication samples | | | | | | | |
| --- | --- | --- | --- | --- | --- | --- | --- | --- | --- | --- | --- | --- | --- | --- | --- | --- | --- |
| Threshold at *p*-value (*p*^*^) | PGC-BP | |  | PGC-MDD | |  | PGC-SCZ | |  | PGC-BP | |  | PGC-MDD | |  | PGC-SCZ | |
|  | #SNPs^a^ | p^b^ |  | #SNPs^a^ | p^b^ |  | #SNPs^a^ | p^b^ |  | #SNPs^a^ | p^b^ |  | #SNPs^a^ | p^b^ |  | #SNPs^a^ | p^b^ |
| 0.005 | 1422 | 0.94 |  | 1155 | 0.40 |  | 1778 | 0.008 |  | 1411 | 0.43 |  | 1158 | 0.25 |  | 1786 | 0.51 |
| 0.01 | 2458 | 0.90 |  | 2132 | 0.38 |  | 2899 | 0.03 |  | 2438 | 0.34 |  | 2130 | 0.98 |  | 2883 | 0.34 |
| 0.05 | 7958 | 0.89 |  | 7759 | 0.56 |  | 8707 | 0.01 |  | 7954 | 0.97 |  | 7743 | 0.50 |  | 8655 | 0.48 |
| Abbreviation: PGC, the Psychiatric Genomics Consortium; BP, bipolar disorder; MDD, major depressive disorder; SCZ, schizophrenia.  ^a^ No. of SNPs having *p*< *p*^*^ & low LD (*r*^2^<0.2). ^b^ *p*-value was calculated using Wilcoxon rank-sum test to test the differences of genetic risk scores between BP-II patients and healthy controls. | | | | | | | | | | | | | | | | | |

**
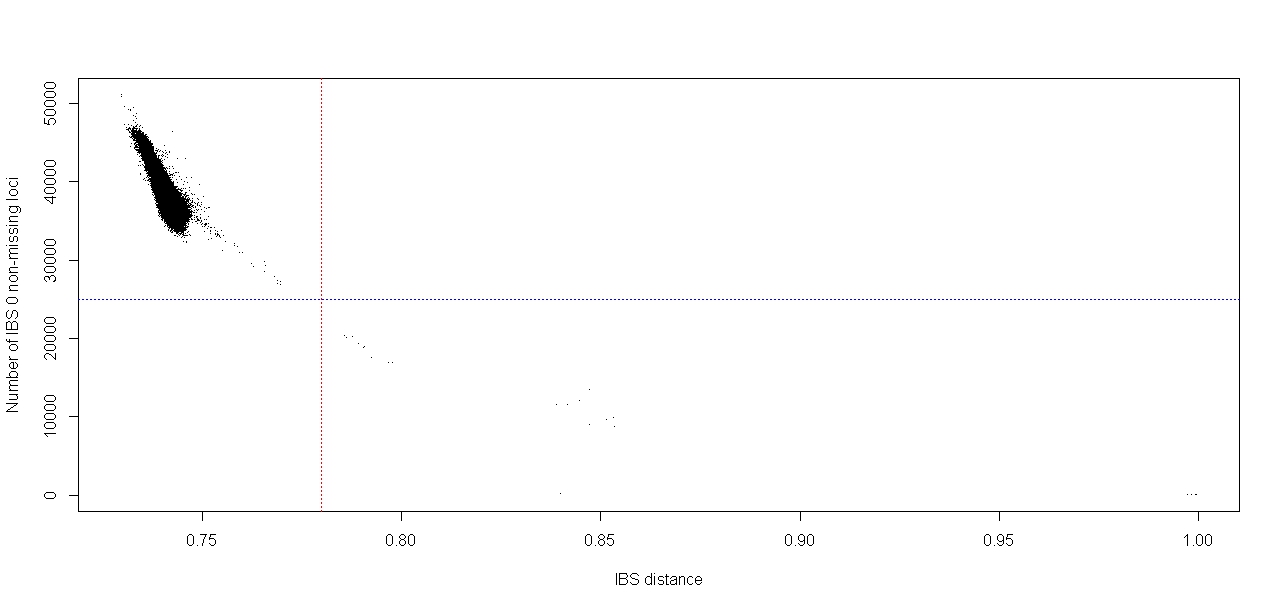
**

**Figure S1. Clustering of kinship data in the discovery samples**


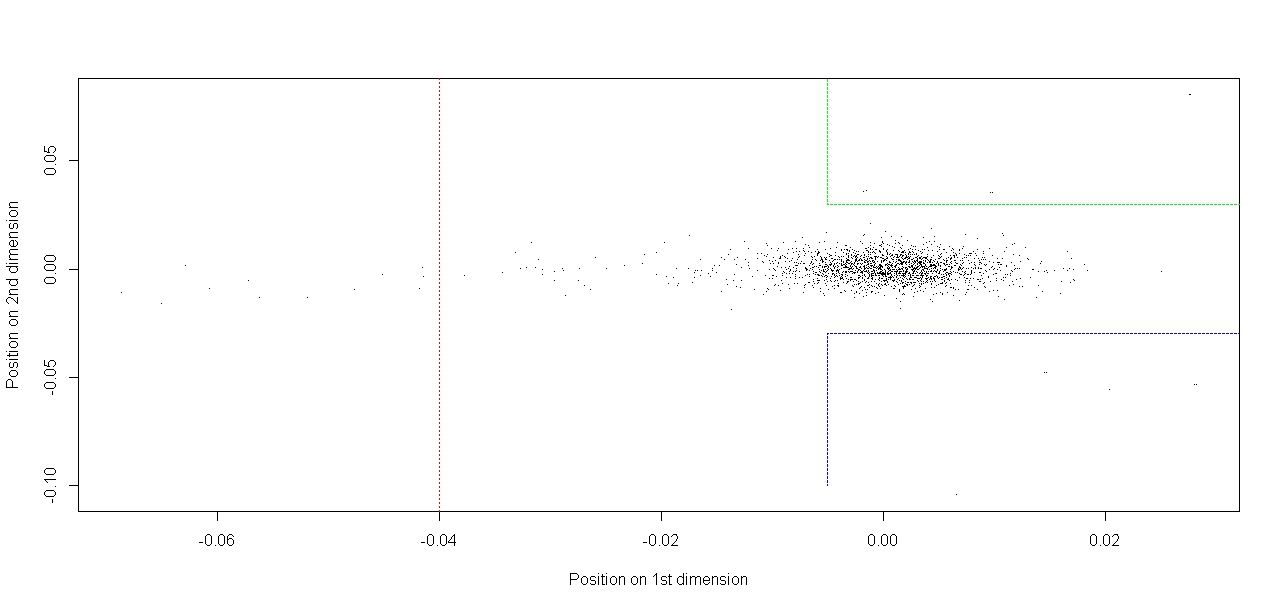


**Figure S2. Clustering of population stratification data in the discovery samples**


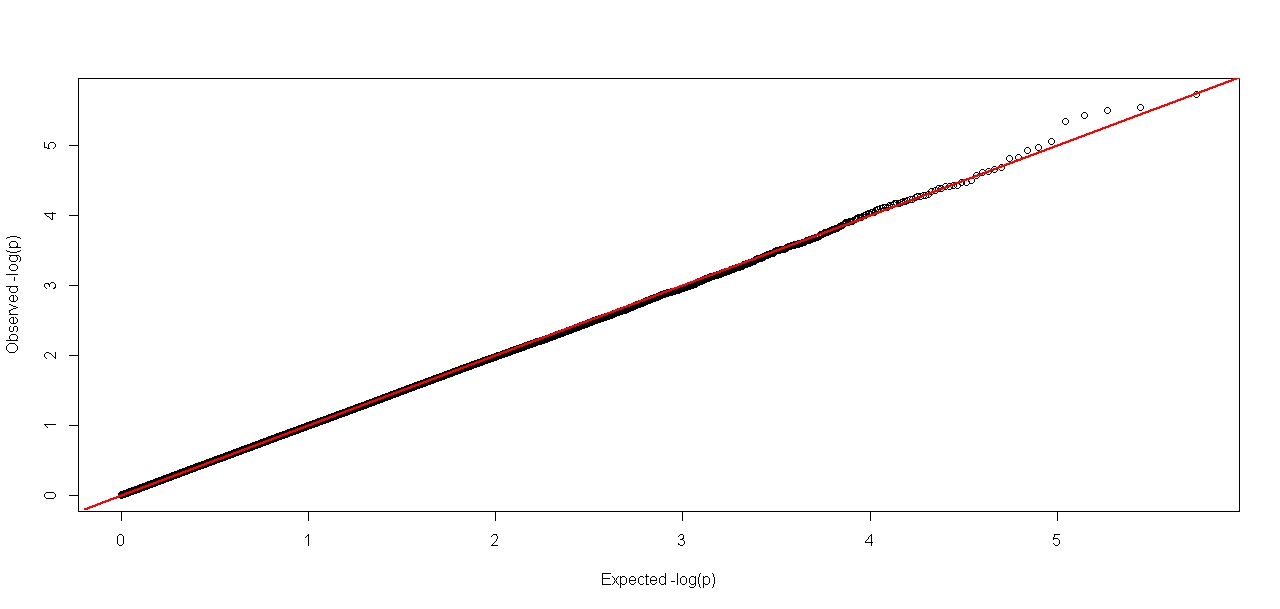


**Figure S3. Quantile-Quantile plot of genome-wide associations (GWA) under additive model with genomic inflation factor of 0.98 in our Taiwanese Han samples**


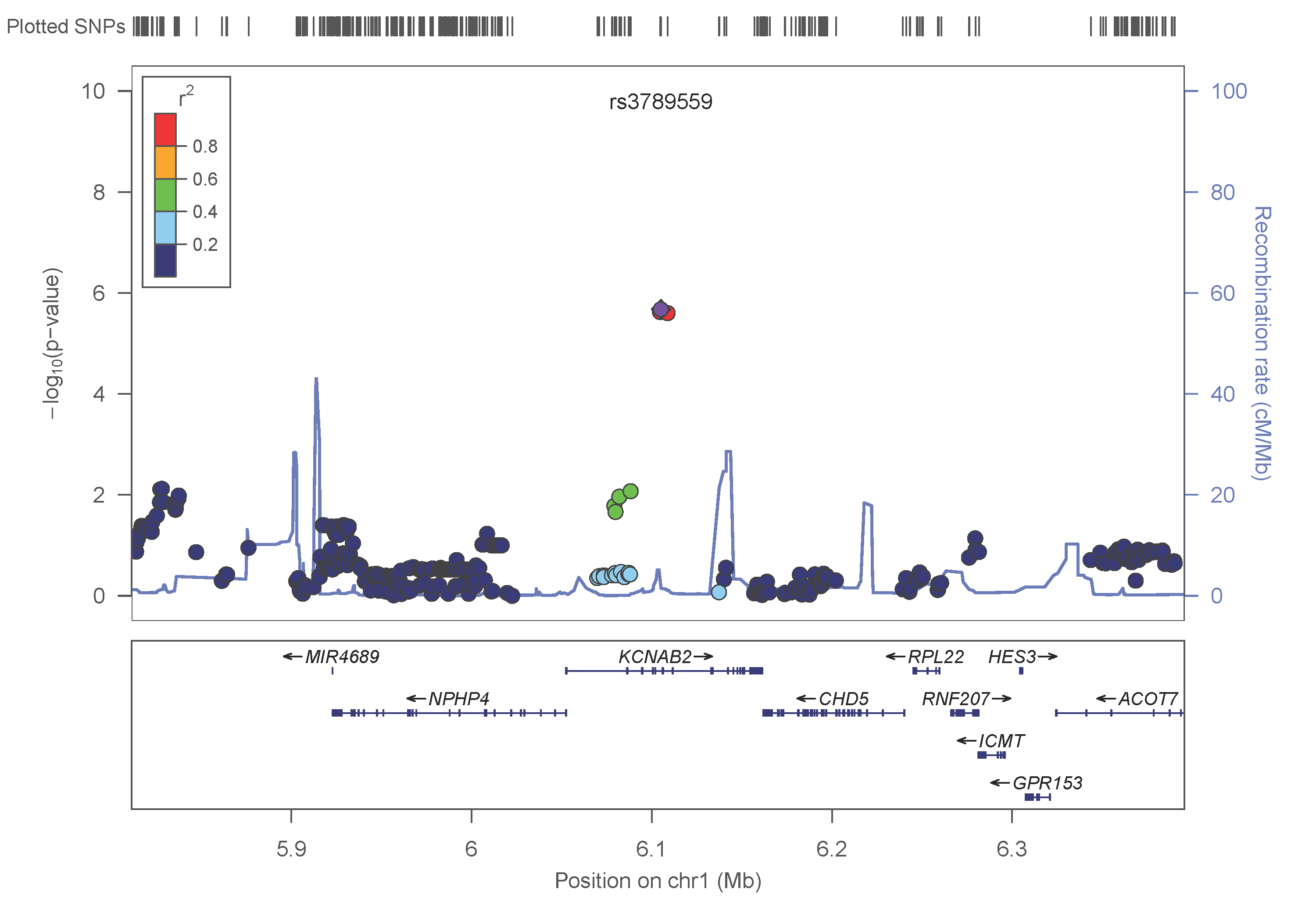


**Figure S4.** **Gene plot of markers within *KCNAB2* gene on chromosome 1**


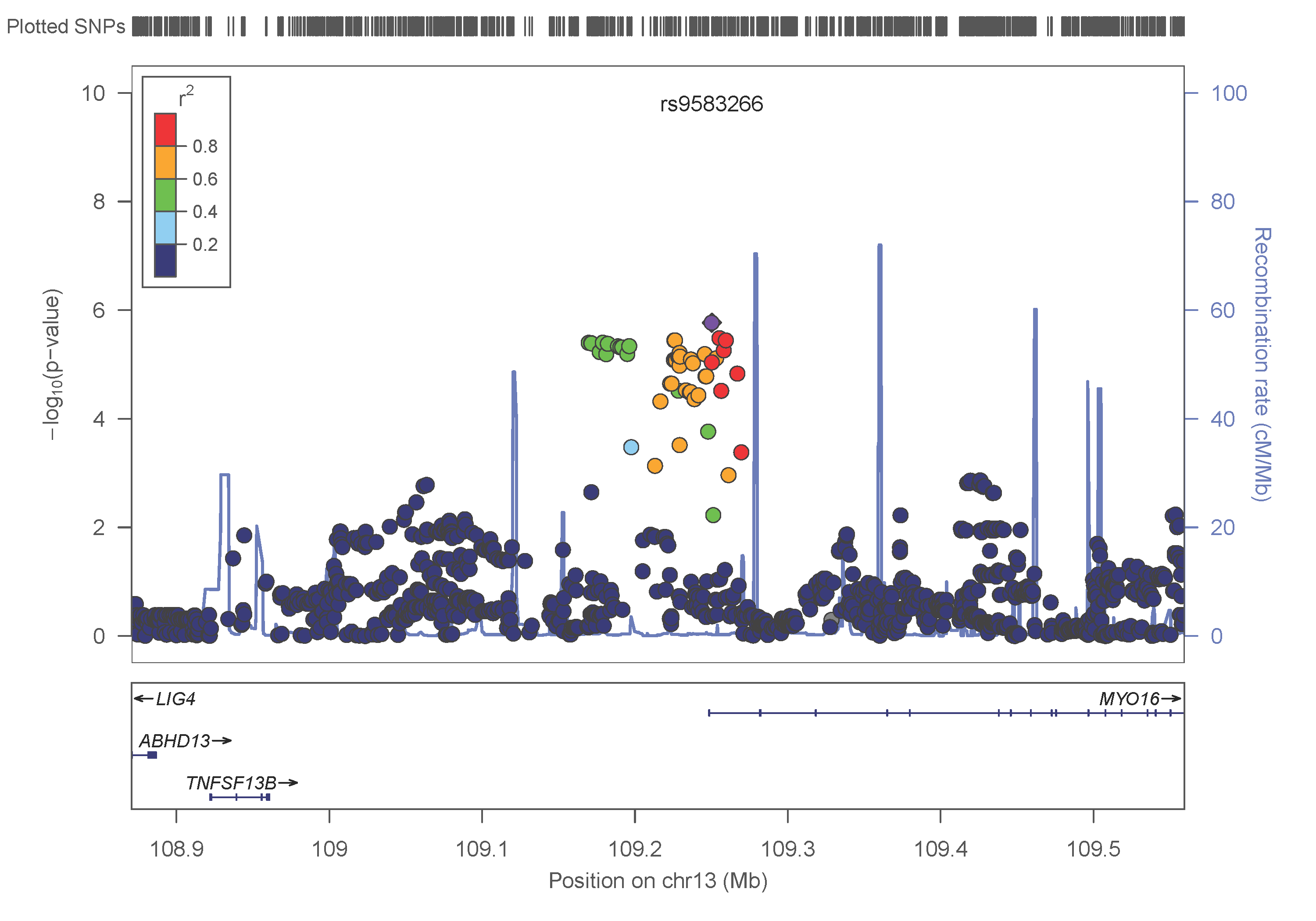


**Figure S5. Gene plot of markers within *MYO16* gene on chromosome 13**


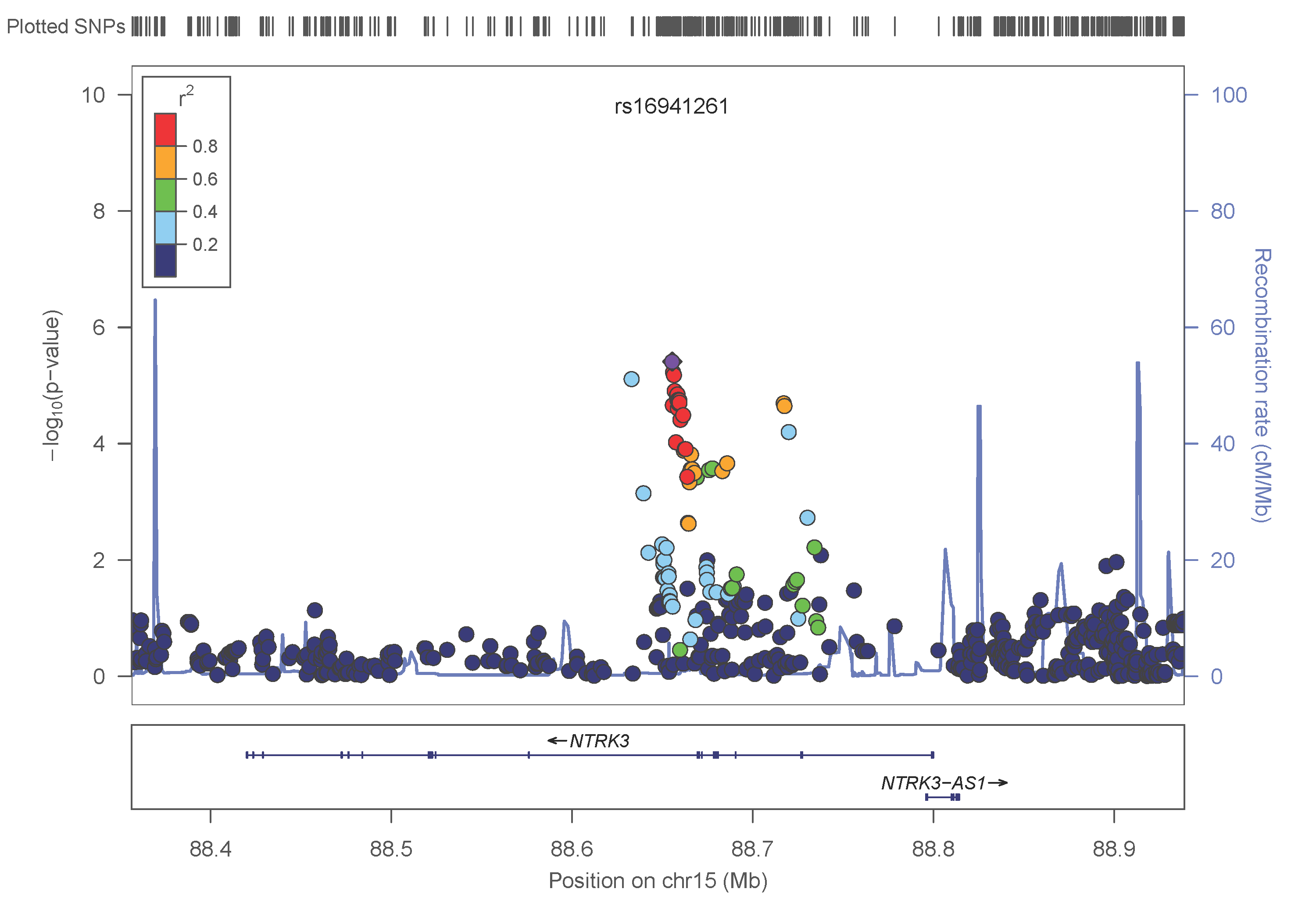


**Figure S6.** **Gene plot of markers within *NTRK3* gene on chromosome 15**


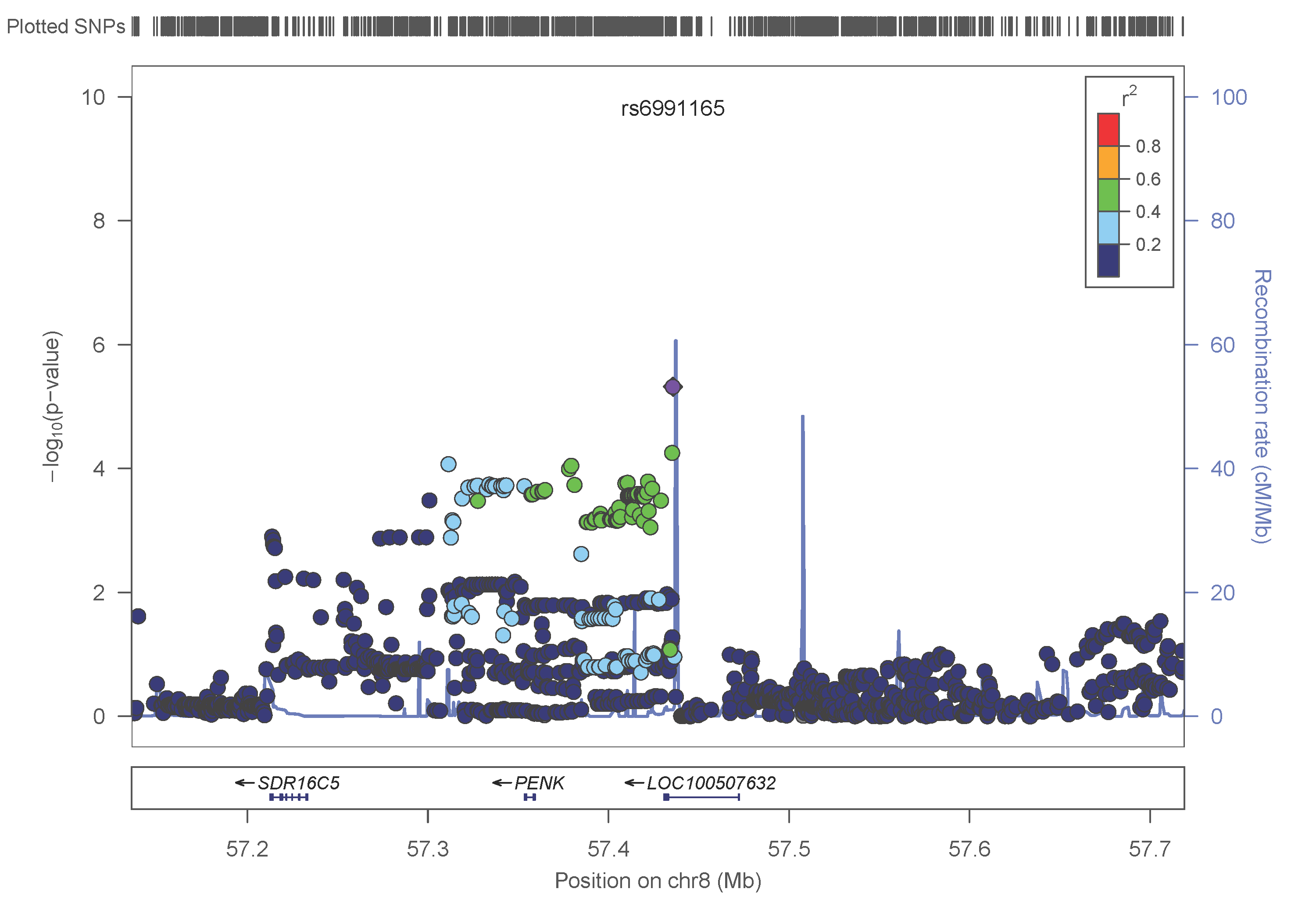


**Figure S7.** **Gene plot of markers within *LOC100507632* gene on chromosome 8**

**
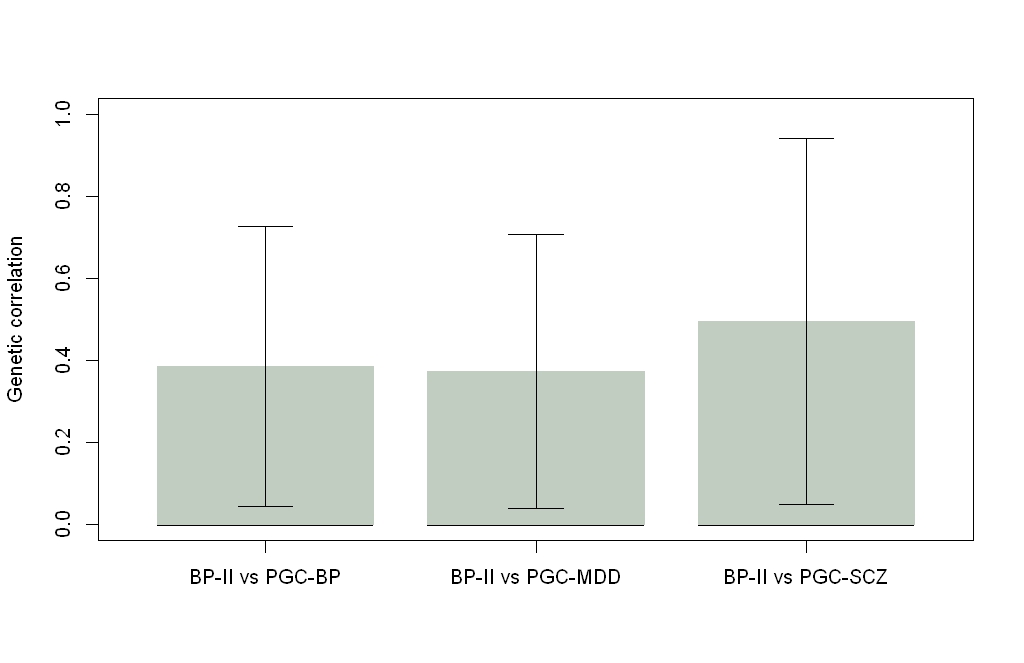
**

**Figure S8. Genetic correlation estimated from corss-trait LD score regression between our BP-II and other psychiatric disorders using PGC datasets of BP, MDD, and SCZ.** The horizontal axis indicates pairs of phenotypes, and the vertical axis indicates genetic correlation. Error bars represent standard errors.
